# High content image analysis reveals function of miR-124 upstream of Vimentin in regulating motor neuron mitochondria

**DOI:** 10.1038/s41598-017-17878-x

**Published:** 2018-01-08

**Authors:** Tal Yardeni, Raquel Fine, Yuvraj Joshi, Tal Gradus-Pery, Noga Kozer, Irit Reichenstein, Eran Yanowski, Shir Nevo, Hila Weiss-Tishler, Michal Eisenberg-Bord, Tal Shalit, Alexander Plotnikov, Haim M. Barr, Eran Perlson, Eran Hornstein

**Affiliations:** 1 Department of Molecular Genetics, Weizmann Institute of Science, Rehovot, 7610001 Israel; 2 Department of Physiology and Pharmacology, Sackler Faculty of Medicine, Tel Aviv University, Tel Aviv, Israel; 3 HTS unit, G-INCPM, Weizmann Institute of Science, Rehovot, 7610001 Israel; 4 Bioinformatics unit, G-INCPM, Weizmann Institute of Science, Rehovot, 7610001 Israel; 5 Present Address: Center for Mitochondrial and Epigenomic Medicine, Children’s Hospital of Philadelphia, Philadelphia, Pennsylvania USA

## Abstract

microRNAs (miRNAs) are critical for neuronal function and their dysregulation is repeatedly observed in neurodegenerative diseases. Here, we implemented high content image analysis for investigating the impact of several miRNAs in mouse primary motor neurons. This survey directed our attention to the neuron-specific miR-124, which controls axonal morphology. By performing next generation sequencing analysis and molecular studies, we characterized novel roles for miR-124 in control of mitochondria localization and function. We further demonstrated that the intermediate filament Vimentin is a key target of miR-124 in this system. Our data establishes a new pathway for control of mitochondria function in motor neurons, revealing the value of a neuron-specific miRNA gene as a mechanism for the re-shaping of otherwise ubiquitously-expressed intermediate filament network, upstream of mitochondria activity and cellular metabolism.

## Introduction

microRNAs (miRNAs) are endogenous non-coding RNAs that facilitate sequence-dependent posttranscriptional silencing, playing pivotal roles in brain development and neuronal function^1–3^. miRNA activity is required for motor neuron survival^4^ and broad dysregulation of miRNA biogenesis is associated with Amyotrophic Lateral Sclerosis (ALS)^4–8^. Several miRNA genes have already been suggested to play critical roles in motor neurons, including miR-155^5^, miR-206^6^, miR-338^9^, miR-9^4^ and miR-218^10–12^.

Mitochondria are cytoplasmic organelles implicated in ATP synthesis, calcium ion homeostasis and apoptotic cascades. Mitochondria are abundant in neurons, because of their high energetic demands^13^. Accordingly, mitochondria regulate axonal growth^14,15^. Mitochondrial dysfunction is implicated in neurodegeneration, including in Alzheimer’s Disease (AD)^16^, Parkinson’s Disease (PD)^17^, Huntington’s Disease (HD)^8^ and ALS^18^. For example, in ALS and in PD, impaired axonal transport causes abnormal accumulation of mitochondria in proximal axons^17,19^.

Functional interconnections between miRNAs and mitochondria were suggested by the existence of mature and precursor miRNAs in purified mitochondria^20–22^ and by the involvement of mitochondrial activity in RNA induced silencing complex (RISC) assembly and miRNA-mediated silencing^23,24^. Furthermore, miRNAs control nuclear-encoded mitochondrial proteins^25^ and mitochondrial metabolism^26,27^. Intriguingly, in neurons miRNAs regulate mitochondrial electron transfer^28^, respiration^29^ and pro-apoptotic mitochondrial cytochrome c release^30^.

In the current report we implemented high content image analysis for investigating the impact of miRNAs on primary motor neurons. We demonstrate that miR-124 overexpression impacts motor neuron morphology and mitochondrial activity. By performing next generation sequencing and molecular studies, we identified the intermediate filament Vimentin (Vim) as an important target of miR-124 in this new pathway. Vim is known to physically associate with mitochondria in different cell types, controlling its position and metabolic activity^31,32^. We show that a new miR-124-Vim pathway regulates mitochondria function and localization, at least in part via control of axonal transport. Our study reveals that Vim functions as a regulator of mitochondrial activity in motor neurons, downstream of miR-124.

## Results

Here, we tested the impact of miRNAs on motor neuron morphology and function, which led us to discover a new pathway for regulation of mitochondria activity, downstream of miR-124.

We first calibrated a transfection system for miRNA in primary mouse motor neurons^33^. We isolated motor neurons from embryonic spinal cords, following^34^, and transfected a hematopoietic miRNA, miR-142, or a scrambled dsRNA sequence at a concentration of 0.1 ng/µl or 0.5 ng/µl. We then measured miRNA levels in cell lysates by quantitative real time PCR (qPCR), 72 hours (hrs) post transfection (Sup. Figure 1a). Transfected miR-142 repressed the expression of a known target, Cofilin 2 (Cfl2)^35^ by 70% after 72 hrs., relative to untransfected or scrambled mimic controls (Sup. Figure 1b). Thus, an exogenous, transfected, miRNA mimic functionally silences endogenous targets in primary mouse motor neurons.

**Figure 1:**
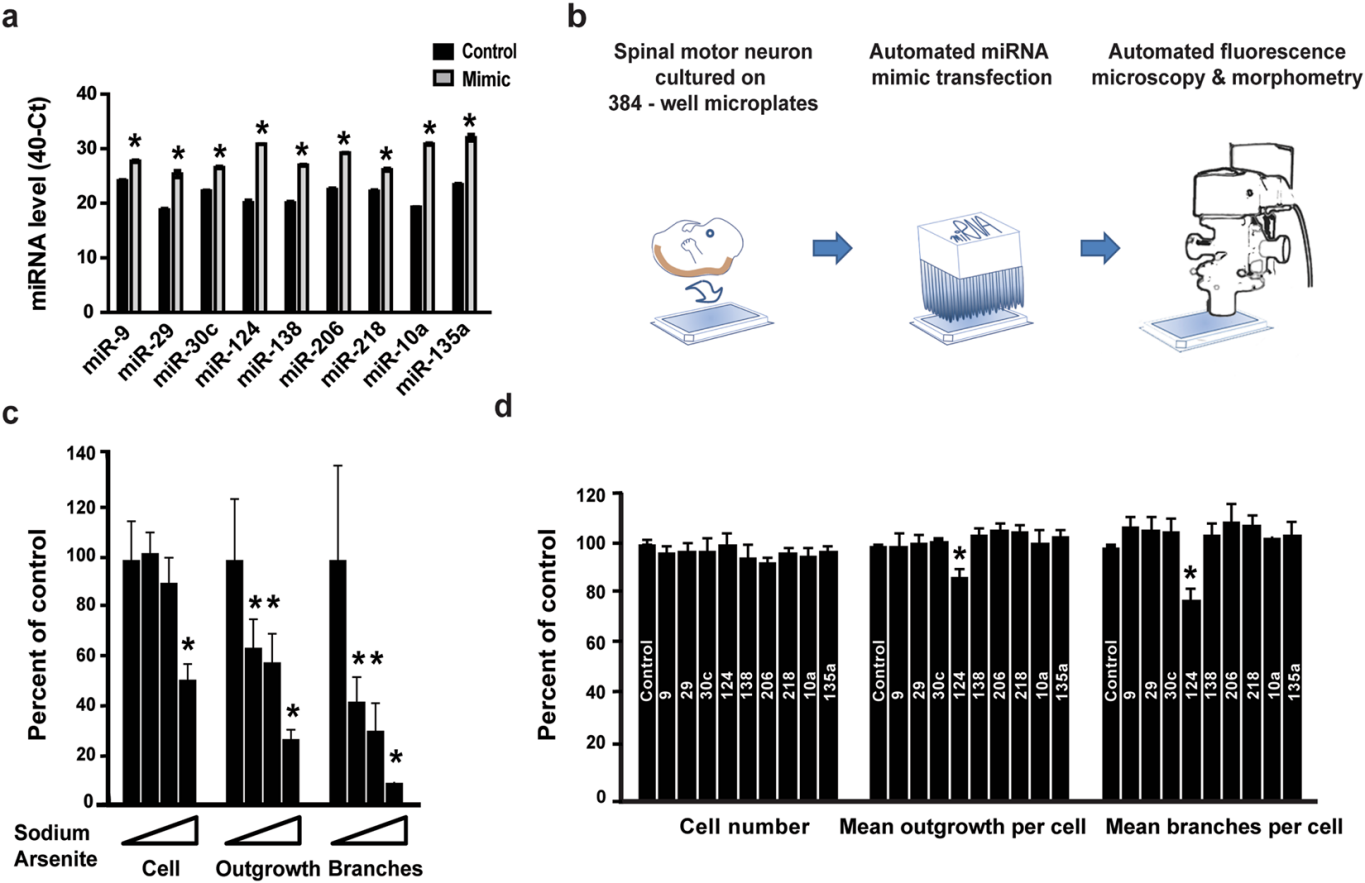
High content image analysis reveals the impact of miR-124 on primary motor neuron morphology. (**a**) Values for nine individual miRNAs, all transfected at 0.5 ng/µl, to mouse primary motor neurons. miRNA expression levels displayed on the Y-axis as 40 minus qPCR cycle threshold (40-Ct), on a Log_2_ scale. All miRNAs were significantly overexpressed. (**b**) A diagram describing the method: Spinal motor neurons were isolated from E13.5 mouse embryos and seeded on a 384 multiwell plate. Culture was transfected with different miRNA mimics using Bravo automated liquid handling robot. 72 hrs later, cells were fixed, stained with anti Tuj1 antibody and DAPI. Two fluorescent micrographs were captured per well (ImageXpress Micro and MetaXpress2 software, Molecular Devices). (**c**) Cell numbers (Cell), neurite outgrowth per cell (outgrowth) and number of branches per cell (branches), were quantified with serial doses of the stress-inducing agent, Sodium Arsenite (15, 30 and 60 µM, for 60 minutes). See Methods and Sup. Figure 2. (**d**) None of the nine miRNAs tested influenced cell numbers. miR-124 was the only miRNA to reduce mean axonal outgrowth per cell and mean number of branches per cell. 500 Tuj1+ neurons quantified per field, 2 fields/well and 6 wells per treatment in five independent experimental repeats. Data collected from >30,000 Tuj1+ neurons per treatment. Averages $\pm$ SEM, Student’s t-test. *P-value < 0.05.

Then we selected nine different miRNA candidates for investigation, including miR-9^36^, miR-29^37^, miR-135^38^, miR-138^39^, miR-30c^40^, miR-124^41^, miR-218^10–12^, miR-10a^42^ and miR-206^6^. A qPCR study revealed that the synthetic mimics upregulated miRNA expression, 72 hrs after transfection (Fig. 1a).

We then cultured primary motor neurons in 384-multiwell plates, transfected miRNA mimics and employed an ImageXpress Micro XLS Widefield High-Content Analysis System for measuring cell numbers, neurite outgrowth and number of branches per cell (Fig. 1b and Sup. Figure 2). By applying Sodium Arsenite (NaAsO_2,_ 15 or 30 or 60 µM, for 60 minutes), a chemical that induces mitochondrial oxidative stress and leads to neurite outgrowth abnormalities^43,44^, we demonstrated the system’s ability to sense morphometric abnormalities (Fig. 1c).

We tested the impact of the nine different miRNA candidates on neuronal morphology. Under all experimental conditions, cell numbers were unchanged, indicating that there was no cell death. While most miRNAs tested did not alter neuronal morphology, miR-124 overexpression caused a significant decrease in axon outgrowth and in the number of branches (Fig. 1d).

**Figure 2:**
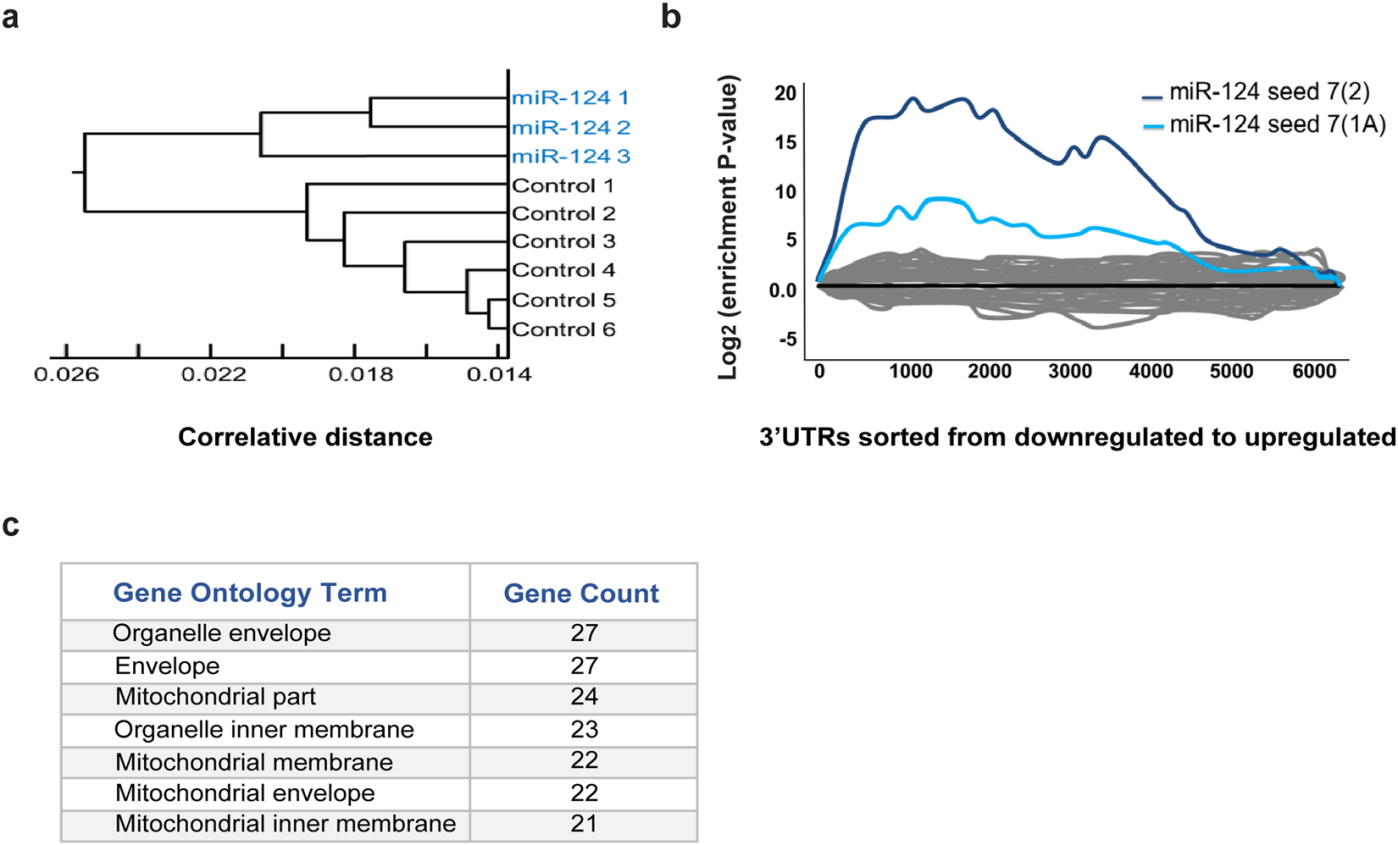
Unbiased bioinformatics analysis of miR-124 targets. Next generation sequencing was performed on RNA extracted from primary motor neurons transfected with either miR-124 mimics (N = 3) or control mimics (N = 6). (**a**) A dendrogram depicting the hierarchical clustering of Pearson correlation coefficients for global gene expression from miR-124 overexpressing motor neurons (blue) and controls (black). (**b**) Enrichment landscape plot for all 876 7mer motifs complementary to canonical mouse miRNA seed regions, gained by Sylamer analysis^45^. Sorted 6500 mRNAs expressed in primary motor neurons, ranked from down- to up-regulated after overexpressing of miR-124, or control mimic. Assessment of over- and under-represented miRNA recognition sequences (seed-matches) for all known miRNAs, identified two enriched motifs, both matching the miR-124 ‘seed’ sequence (blue, 7mer–2; light blue, 7mer-1A). (**c**) Top seven terms (by gene count) from gene ontology (GO) analysis on all mRNAs significantly up- or down- regulated following miR-124 overexpression (corrected P-value < 0.05), using database for annotation, visualization and integrated discovery (DAVID) software^46^. This analysis revealed an enrichment for mitochondrial-related genes and is further described in Table 1.

To uncover the underlying molecular mechanism responsible for miR-124 activity, we performed transcriptome profiling, using next generation sequencing (NGS). Total RNA was extracted from primary motor neurons and 3′ cDNA libraries were constructed and sequenced. Hierarchical clustering analysis of mRNA expression depicted a unique expression profile for neurons that overexpressed miR-124, which was distinguishable from cells transfected with scrambled control oligos (dendrogram of Pearson correlation coefficient, Fig. 2a).

Sylamer analysis^45^ of 6500 expressed mRNAs, from neurons expressing scrambled control mimics or miR-124, uncovered two enriched motifs, which matched the miR-124 ‘seed’ sequence. However, such enrichment was not evident for any other miRNA gene (Fig. 2b). We conclude that miR-124 overexpression had a widespread and specific impact on motor neuron mRNA expression profile.

Gene ontology (GO) analysis was carried out for approximately 1100 mRNAs that were significantly up- or downregulated, following miR-124 overexpression (corrected P-value < 0.05), using DAVID^46^. Intriguingly, many of the gene ontology terms highlighted the potential relevance of mitochondria-related structure or function in response to miR-124 (Table 1, Fig. 2c). We therefore further hypothesized that miR-124 plays a role in the regulation of mitochondrial functions.

**Table 1:** Gene ontology (GO) terms for changes in motor neuron mRNA expression after miR-124 overexpression.

Gene Ontology term	genes count	P-value
Organelle envelope	27	1.80E-05
Envelope	27	1.90E-05
Mitochondrial part	24	2.30E-04
Organelle inner membrane	23	1.80E-07
Mitochondrial membrane	22	1.00E-05
Mitochondrial envelope	22	2.50E-05
Mitochondrial inner membrane	21	1.30E-06
Inorganic cation transmembrane transporter activity	16	8.40E-09
Monovalent inorganic cation transmembrane transporter activity	14	4.70E-09
Hydrogen ion transmembrane transporter activity	13	2.40E-08
Redox-active center	8	4.60E-06
Cell redox homeostasis	8	1.00E-04
Thioredoxin-like	6	3.40E-04
Thioredoxin-like	6	3.40E-04
Disulphide isomerase	5	1.20E-06
Domain:Thioredoxin 2	5	9.30E-06
Domain:Thioredoxin 1	5	9.30E-06
Thioredoxin-like subdomain	5	2.50E-05
Thioredoxin, conserved site	5	7.80E-04
Thioredoxin domain	5	9.10E-04
Oxidoreductase activity, acting on heme group of donors, oxygen as acceptor	5	6.90E-04
Oxidoreductase activity, acting on heme group of donors	5	6.90E-04
Cytochrome-c oxidase activity	5	6.90E-04
Heme-copper terminal oxidase activity	5	6.90E-04
PIRSF001487:protein disulfide-isomerase	4	1.90E-04
Protein disulfide isomerase activity	4	3.00E-04
Intramolecular oxidoreductase activity, transposing S-S bonds	4	3.00E-04
Intramolecular oxidoreductase activity, interconverting keto- and enol-groups	4	4.50E-04
Intramolecular oxidoreductase activity	4	3.10E-02
Protein disulphide isomerase	3	1.70E-03

Top 30 Gene ontology (GO) term analysis of up- or downregulated mRNAs after miR-124 overexpression (corrected P-value < 0.05). Table columns include DAVID^46^ term names, number of genes counted in each category and p-value for the statistical significance of the category. There is noticeable overrepresentation of gene ontology terms related to mitochondria structure or function in response to miR-124.

**Figure 3:**
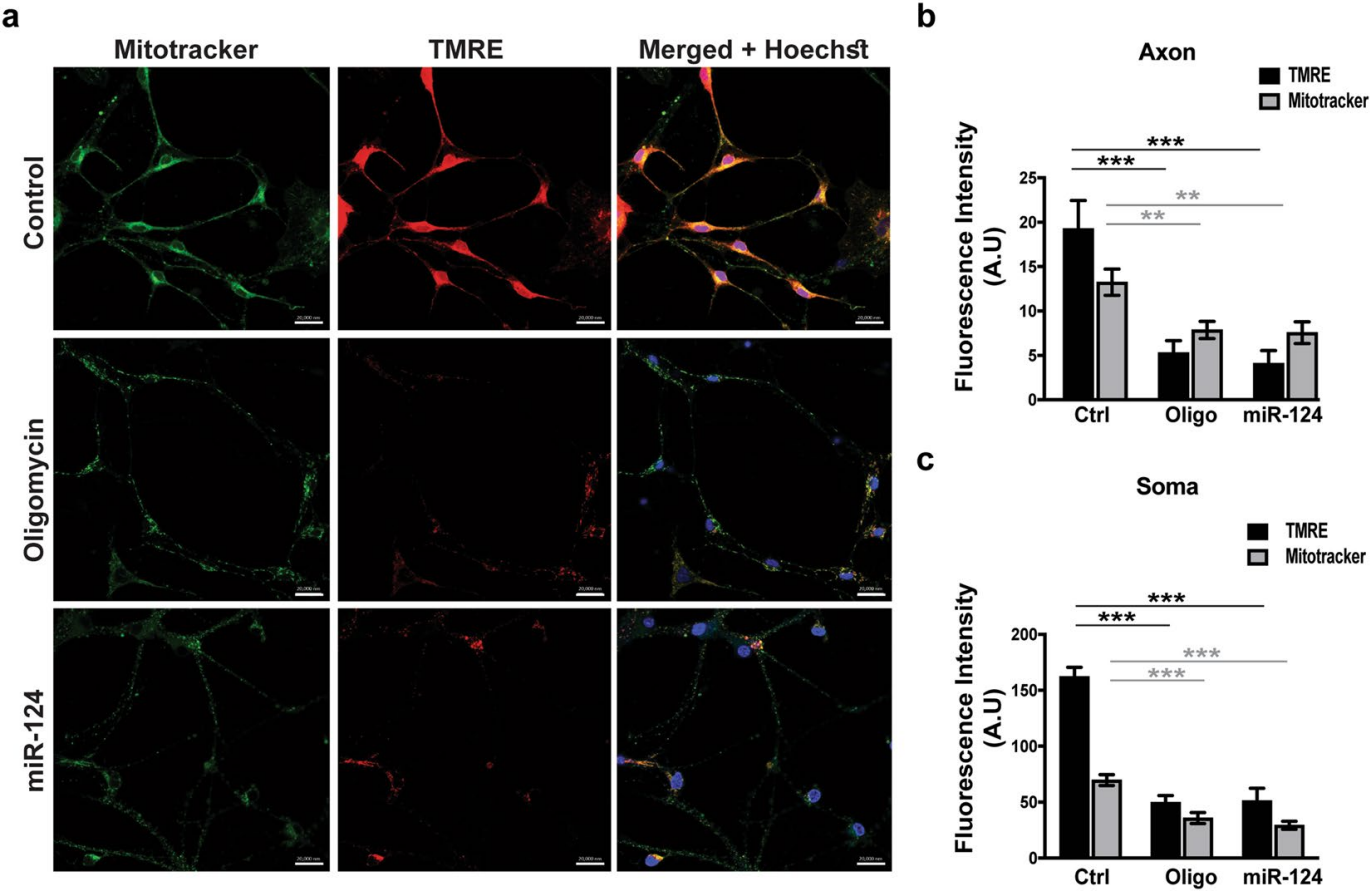
miR-124 overexpression impacts mitochondrial localization and function. (**a**) Confocal fluorescent micrographs of primary motor neurons, 72 hrs post transfection with scrambled control or miR-124 mimics, stained with MitoTracker Deep Red FM (green) and TMRE (red) to depict functional mitochondria. Merged signal (yellow) relative to nuclei (blue, Hoechst), scale bars, 20 μm. Oligomycin A (1 µM) or miR-124 overexpression diminished mitochondrial activity. Bar graphs quantification of fluorescent signal intensity in (**b**) axons and (**c**) soma. Quantification of 3 random positions per compartment; 9 neurons per condition; Data collected from three replicates and the study performed in > four independent experimental repeats. Averages ± SEM, one way ANOVA with post hock Neuman-Keuls. *P-value < 0.05, **p < 0.01, ***p < 0.001.

We studied mitochondria function and  intracellular position by employing Mitotracker Deep Red FM, a mitochondrion-selective dye that accumulates in active mitochondria. We also used in parallel Tetramethylrhodamine ethyl ester (TMRE), which is a fluorescent indicator of mitochondria membrane potential. Both TMRE and Mitotracker signals were dampened by the activity of Oligomycin A^47^, a benchmark inhibitor of mitochondrial ATP synthase. Interestingly, miR-124 overexpression inhibited TMRE and Mitotracker signals reminiscent of Oligomycin A, leading us to conclude that miR-124 regulates mitochondrial function (Fig. 3).

Confocal immunofluorescence of the mitochondrial marker, ATP5A, and an ultrastructural study with transmission electron microscopy (TEM), revealed depletion of mitochondria in primary motor neuron axons, which overexpressed miR-124, relative to controls. (Sup. Figures 3, 4). Therefore, miR-124 regulates mitochondria position and activity in motor neurons.

**Figure 4:**
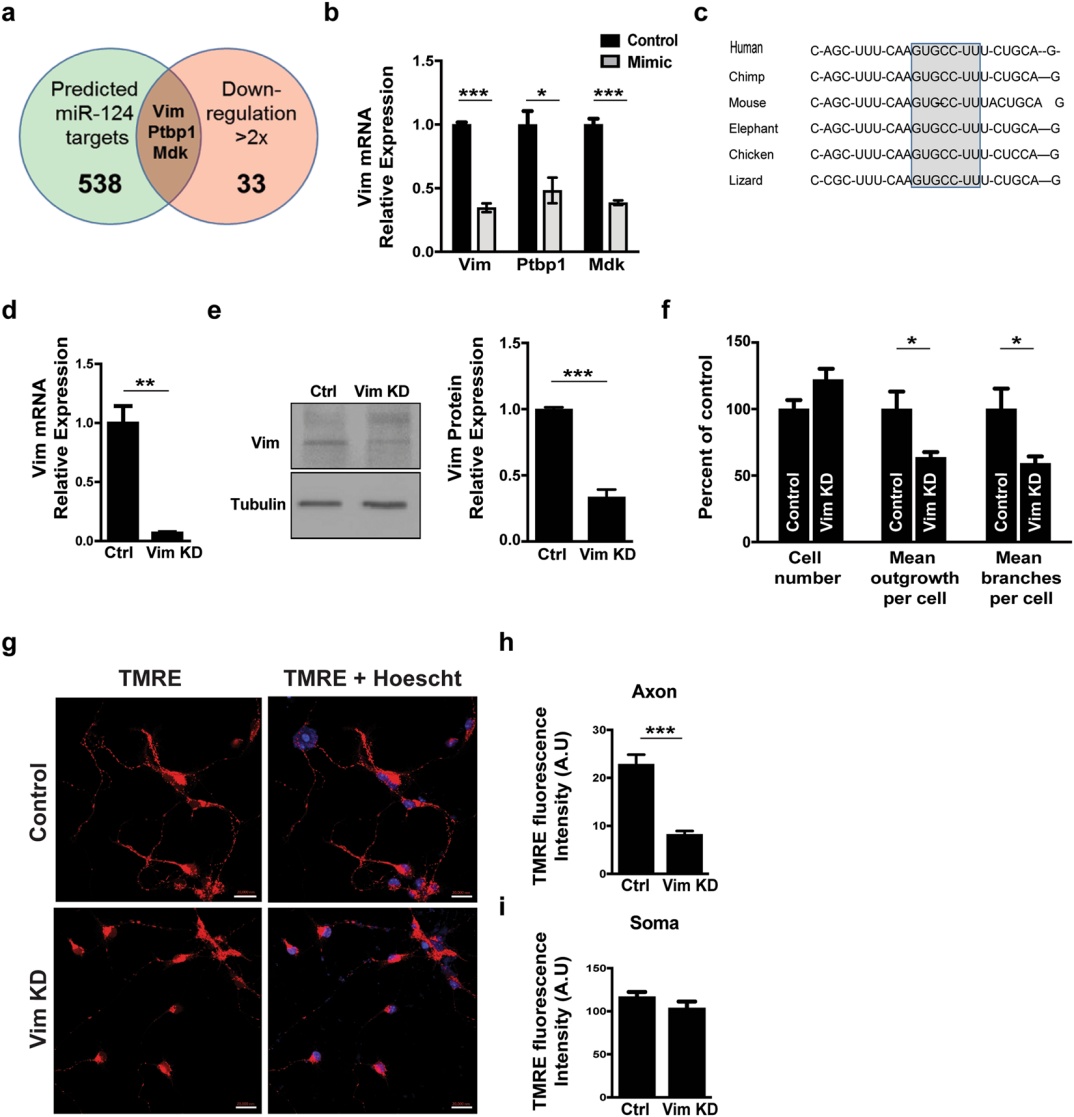
Vimentin regulates neuron morphology and mitochondria function (**a**) Venn diagram of predicted miR-124 targets (TargetScan) and transcripts that were experimentally repressed >2 fold by miR-124 overexpression in primary motor neurons, relative to control conditions. (**b**) qPCR analysis of the three mRNAs that obeyed both criterions: Vim, Ptbp1 and Mdk, in primary motor neurons transfected with miR-124 oligos (N = 3), relative to controls (N = 3). (**c**) miR-124 recognition element at Vim 3′UTR is conserved in vertebrate species. Knockdown of Vim by shRNA lentiviruses^53^ downregulated (**d**) Vim mRNA and (**e**) VIM protein expression, relative to non-targeting shRNA controls. A full view of the Western blot is in Sup. Figure 6. (**f**) High content image analysis of cell numbers, mean axonal outgrowth per cell and mean number of branches per cell. 100 Tuj1+ neurons quantified per field, 8 fields/well, 6 replicates per treatment. Data collected from >24,000 Tuj1+ neurons per treatment. (**g**) Confocal fluorescent micrographs of primary motor neurons, 72 hrs. post transduction with scrambled-shRNA or Vim-shRNA, stained with TMRE (red) and nuclei (blue, Hoechst). Transduction efficacy >80%. Scale bars, 20 μm. Bar graphs quantification of fluorescent signal intensity in (**h**) axons and (**i**) soma. Quantification of 3 random positions per compartment; 9 neurons per condition. Data from three replicates and the study performed in three independent experimental repeats. All graphs present averages $\pm$ SEM, Student’s t-test. *P-value < 0.05, **p < 0.01, ***p < 0.001.

To characterize the targets and pathways that are regulated by miR-124, we intersected the list of mRNAs that were repressed more than twofold by miR-124 overexpression in the NGS data, with the list of predicted miR-124 targets (TargetScan^48^). Three genes that harbor conserved miR-124 binding sites, were also downregulated more than twofold by miR-124 overexpression, namely, Polypyrimidine Tract Binding Protein 1 (Ptbp1, MGI:97791), Midkine (Mdk, MGI:96949) and Vimentin (Vim, MGI:98932; Fig. 4a). qPCR study validated that miR-124 overexpression inhibited Vim to ~1/3 its levels compared with control cells that were treated with scrambled oligos. Ptbp1, an established miR-124 target^49^, and Mdk were downregulated to ~1/2 their expression level (Fig. 4b).

Interestingly, Vim is a known regulator of mitochondria localization and activity^31,32^, with miR-124 binding sites that are conserved across several vertebrate species (Fig. 4c). Furthermore, we identified molecular evidence for direct interactions of miR-124 with the target Vim, in Argonaute CLIP studies^50,51^ and a Vim 3′UTR reporter was inhibited by miR-124 mimics in hepatocellular carcinoma cells^52^.

We sought to inhibit Vim independently of miR-124, and test its effect on cell morphology and mitochondria activity. Lentiviral transduction of primary motor neurons was very efficient (Sup. Figure 5) and allowed us to effectively knockdown Vim by shRNA^53^. Vim shRNA reduced Vim mRNA and protein levels (Fig. 4d,e, Sup. Figure 6a). Accordingly, high content image analysis of Vim knockdown depicted reduction in neurite outgrowth and branching (Fig. 4f), recapitulating miR-124 activity. In addition, Vim knockdown resulted in inhibition of mitochondria activity in axons, but not in the soma (Fig. 4g–i). Differences in the effect of miR-124 on soma may be the result of transfection efficiency. Intriguingly, miR-124 is primarily peri-nuclear^54^. Therefore, the soma compartment may be less amendable to manipulation, whereas, in the axon, where the miRNA is expressed at lower levels, the effects of miR-124 overexpression were consistent across all observations. In conclusion, Vim pheno-copies miR-124 functions, further suggesting that both genes are engaged in the same pathway.

**Figure 5:**
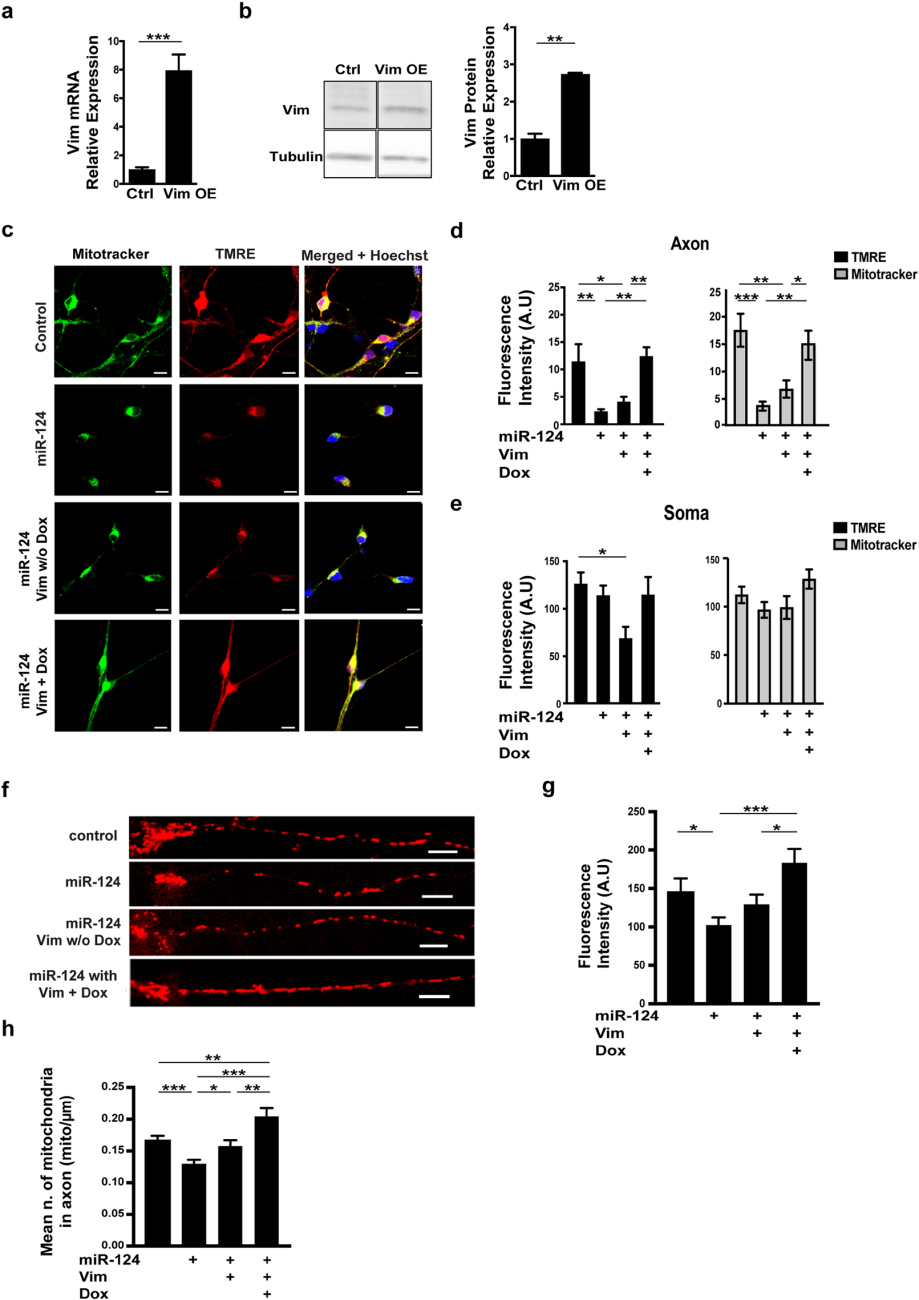
miR-124 regulates mitochondria activity via Vimentin. Primary motor neurons of control, miR-124 overexpression alone or transduced in addition with lentiviral vector for Doxycyclin-dependent expression of Vim (>80% transduction efficacy, transduction 24 hrs. before transfection of miRNA mimics), without or with the chemical inducer (Dox). (**a**) Vim mRNA and (**b**) VIM protein expression without or with Dox. Three experimental repeats, Averages $\pm$ SEM, Student’s *t*-test. A full view of the Western blot is in Sup. Figure 6. (**c**) Confocal fluorescent micrographs of primary motor neurons, stained with MitoTracker (green) and TMRE (red), merged signal (yellow) and nuclei (blue, Hoechst). Scale bars, 20 μm. Bar graphs quantification of fluorescent signal intensity in (**d**) axons and (**e**) soma. Quantification of three random positions per compartment; 9 neurons per condition; Data from three replicates and the study performed in three independent experimental repeats. Averages ± SEM, ANOVA + Duncan’s new multiple range test (MRT) *P-value < 0.05, **p < 0.01, ***p < 0.001. (**f**) Snapshot from mitochondria live imaging along axons of primary motor neurons. Bar graphs quantification of (**g**) axonal mitochondria density/μm and (**h**) mean TMRE intensity in control axons (n = 48), miR-124 overexpression alone (n = 52), miR-124 and Vim without Dox (n = 34) or with Dox (n = 34). Averages $\pm$ SEM, Student’s *t*-test, P-value *p < 0.05, **p < 0.01.

Based on the above observations, we hypothesized that Vim is a novel effector of miR-124 in a pathway that regulates mitochondria function. Therefore, we tested whether upregulating Vim levels is sufficient for recovering mitochondrial activity. Doxycycline-induced expression of Vim, from a lentiviral vector that does not harbor the 3′UTR and hence is not inhibited by miR-124, upregulated Vim mRNA and protein levels (Fig. 5a,b, Sup. Figure 6b). Exogenous Vim was also sufficient to alleviate miR-124-dependent inhibition of mitochondrial function, relative to miR-124 alone (Fig. 5c–e). Furthermore, when counting the numbers of mitochondria in axons, we observed rescue of axonal mitochondria occupancy by exogenous Vim (Fig. 5f–h; We also noted some effect without Dox that is probably due to leaky Vim expression).

**Figure 6:**
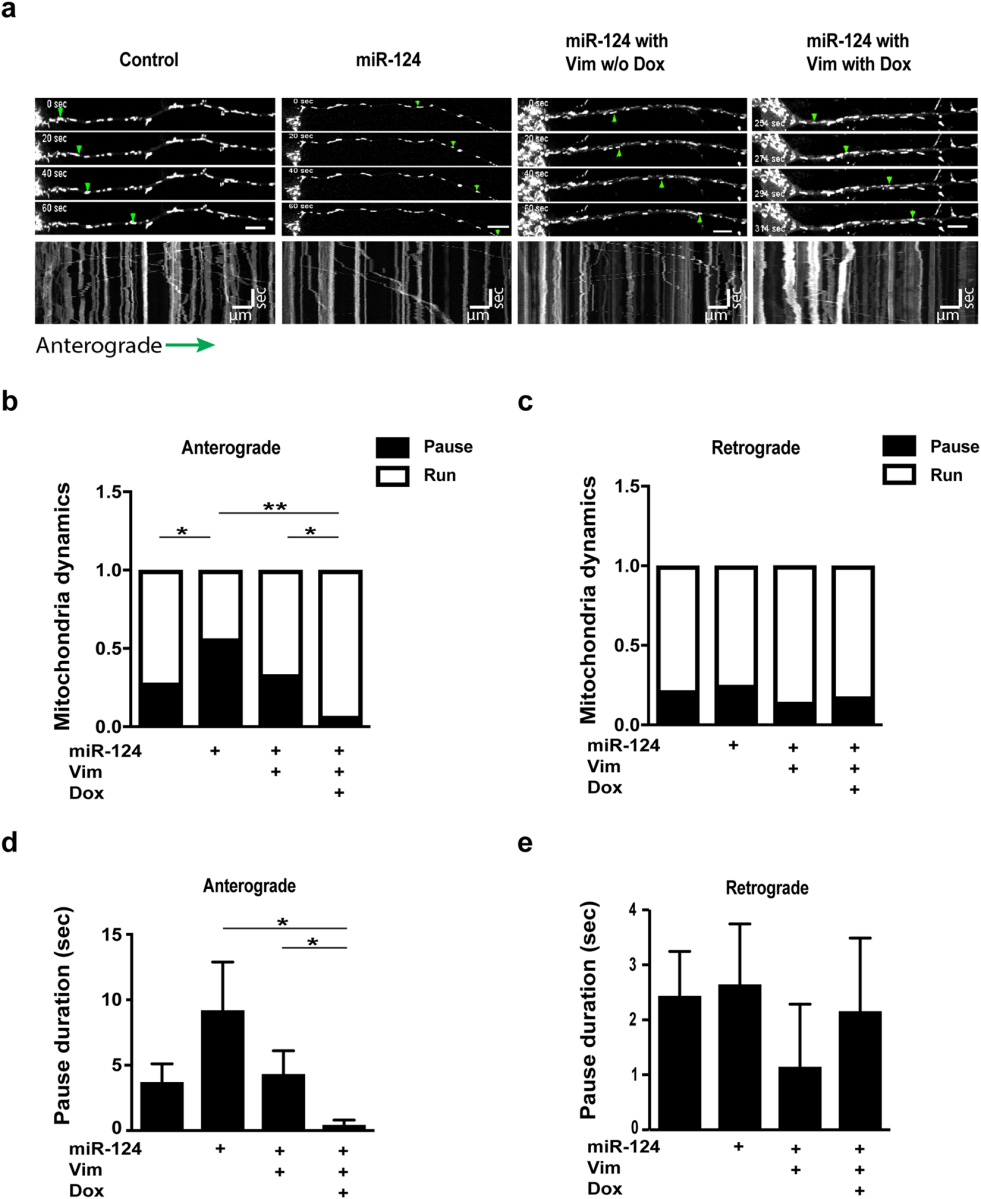
miR-124 and Vimentin co-regulate mitochondria motility in primary motor neurons. Primary motor neurons of control, miR-124 overexpression alone or transduced in addition with lentiviral vector for Doxycycline-dependent expression of Vim (>80% transduction efficacy, performed 24 hrs. before transfection of miRNA mimics), without or with the chemical inducer (Dox). (**a**) Representative captures from mitochondria live imaging and motor axons kymographs, depicting anterograde movement. Green arrows indicate the movement of one representative mitochondrion. Running mitochondria were defined by moving a distance of >10 µm at average speed >0.2 µm/sec. Paused mitochondria are a subpopulation of the running mitochondria, which paused in the same location for ≥3 frames in succession. Proportion of pausing and continuously running mitochondria in (**b**) anterograde or (**c**) retrograde directions. Horizontal scale bar − 10 µm. Vertical scale bar − 60 seconds. Test of proportion statistics, *p < 0.05, **p < 0.01. (**d**) Anterograde or (**e**) retrograde measurement of pause duration (sec). Averages $\pm$ SEM, Student’s *t*-test, P-value *p < 0.05.

Finally, we used live imaging microscopy to test if the new miR-124-Vim axis regulates mitochondria motility in axons. We identified two subtypes of motile mitochondria: mitochondria that were continuously running in an uninterrupted manner along the axon over a distance of >10 µm at average speed >0.2 µm/sec., or mitochondria that displayed intermittent pauses in the same location for ≥3 frames in succession. Intriguingly, miR-124 caused mitochondria to pause more frequently on anterograde route, than in control samples, but did not affect retrograde transport. This may explain the re-distribution of mitochondria after overexpression of miR-124 and relative axonal depletion. Exogenous Vim expression rescued this phenotype and normalized mitochondria running/pausing dynamics (Fig. 6a–c). Furthermore, measurements of pause duration depicted asymmetry in anterograde vs. retrograde (Fig. 6d,e). We noted some effect without Dox that is probably due to leaky Vim expression. Parameters of run length and mean speed, were not changed by miR-124 and Vim (Sup. Figure 7). Therefore, anterograde mitochondria transport is influenced by the number of mitochondria pausing and the typical time interval spent resting during their run. We conclude that the mechanism for control of mitochondria running/pausing propensities in motor neurons involves Vim and miR-124 in a fashion affecting anterograde but not retrograde transport.

Taken together, we characterized a novel pathway in motor neurons downstream of miR-124 in regulation of mitochondria dynamics, distribution and activity. In this pathway, Vim, functions as an important effector of miR-124, revealing a surprising mechanism for controlling energy metabolism in motor neurons by neuronal miRNAs and intermediate filaments.

## Discussion

In the current study, we employed high content image analysis, next generation sequencing and molecular approaches for discovery of a new pathway that is affecting motor neuron axon morphology and mitochondria homeostasis. An initial screen led us to focus on miR-124, which was the only miRNA that exhibited abnormal axonal morphology, out of nine miRNAs that were tested. miR-124 is one of the most abundant miRNA in many neuronal subtypes and is conserved from insects to mammals. miR-124 drives neuronal differentiation, promoting neuroblasts cell-cycle exit^41,55^, neuron-specific alternative splicing and chromatin remodeling via silencing of Ptbp11^49^ and Actin like 6 A/BAF53a^56^, respectively. Furthermore, miR-124 levels remain high in postmitotic neurons, suggesting that it plays a role also in maintenance of the differentiated state of neurons. However, miR-124 roles in motor neuron were not thoroughly investigated.

We demonstrated that miR-124 regulates mitochondrial activity and localization. The cluster of mitochondrial genes that responded to miR-124 overexpression was not particularly enriched in direct targets of miR-124, suggesting indirect regulation. The intermediate filament Vim is a key effector of miR-124 upstream of mitochondria function and localization. Vim knockdown by other means, pheno-copied miR-124 overexpression and an exogenous Vim that does not harbor miR-124 binding sites, rescued the mitochondrial phenotype.

Precise mitochondria localization is critical for maintaining energy and calcium homeostasis in neurons. Appropriate mitochondria localization is essential for neurite outgrowth^57–59^.Minin *et al*., have shown that Vim regulates mitochondria activity and motility^31,32,60^ in other cell types, which is consistent with the discovery of a new miR-124 - Vim axis for unidirectional control of mitochondria transport in axons. miR-124 and Vim asymmetric action is further evocative of kinesins and dynein motor proteins that reciprocally serve anterograde and retrograde transport. However, a direct molecular link to the classic kinesin/dynein system is still missing.

miRNA dysregulation^7,61–63^ and mitochondrial impairments^63–65^, are repeatedly observed in ALS. Our analysis reveals that miR-124 overexpression may be disadvantageous to primary motor neurons, in accordance with reported miR-124 upregulation in late ALS stages in mouse brains^61^ and with injurious miR-124 overexpression in adult hippocampus/prefrontal cortex^66^. In summary, we propose that miR-124 expression levels should be tightly kept within defined margins and that a novel miR-124 - Vim pathway reveals a mechanism, by which miRNAs regulate of axonal mitochondria transport.

## Materials and Methods

### Primary motor neuron culture

All experiments were performed in accordance with relevant guidelines and were approved by the Institutional Animal Care and Use Committee (IACUC) at Weizmann Institute of Science. Primary motor neurons were isolated and cultured as described^34^ with the following modification: spinal cords were dissected from ICR mouse embryos at embryonic day 13.5 (E13.5). Motor neurons were dissociated with papain (2 mg/ml, Sigma), separated through Optiprep gradient (Sigma) and seeded either on 13 mm coverslips (200,000 cells/coverslip, Thermo scientific) or on 384 multiwell plates (7500 cells/well, Griener bio-one, cat# 781091) using a liquid handling device (GNF Systems), pre-coated with poly-ornithine (3 µg/ml) or poly L Lysine (Sigma P4707, 0.002% in Borate buffer 0.1 M ph 8.5, Sigma) and then Laminin (3 µg/ml, Gibco | Thermo Fisher Scientific). Motor neurons were cultured with Neurobasal (Gibco | Thermo Fisher Scientific)/B27 (Gibco | Thermo Fisher Scientific) medium supplemented with 2% horse serum (Sigma), and 1 ng/ml CNTF, 1 ng/ml GDNF (Peprotech) at 37 °C. For live imaging motor neurons were isolated as in^67,68^.

### miRNA mimics transfection

miRNA mimics, were dsRNA oligonucleotides (Integrated DNA Technologies, Inc.), as described in Table 2. dsRNA encapsulated in Neuro9™ nanoparticles (Precision NanoSystems, Inc.)^33^. Mimics (0.5 ng/µl) were transfected 24 hrs after seeding of primary motor neurons manualy or by using Bravo automated liquid handling robot.

**Table 2:** List of synthetic DNA and RNA oligos used in the study.

miRNA mimics, designed as dsRNA oligonucleotides r- RNA bases; r_ * - Phosphorothioated RNA base; m- 2’ O-methyl RNA base		
	Anti- sense mimic	Sense mimic
miR-142-3p	rU*rG*rUrArGrUrGrUrUrUrCrCrUrArCrUrUrUrArUmGmGrA	5SpC3/rUmCrCmArUmArAmArGmUrAmGrGmArAmArCmArCmUrAmCA
miR-9-5p	rU*rC*rUrUrUrGrGrUrUrArUrCrUrArGrCrUrGrUrAmUmGrA	mAmUrAmCrAmGrCmUrAmGrAmUrAmArCmCrAmArAmGA
miR-10a-5p	rU*rA*rCrCrCrUrGrUrArGrArUrCrCrGrArArUrUrUmGmUrG	mCmArAmArUmUrCmGrGmArUmCrUmArCmArGmGrGmUA
miR-29a-3p	rU*rA*rGrCrArCrCrArUrCrUrGrArArArUrCrGrGmUmUrA	mAmCrCmGrAmUrUmUrCmArGmArUmGrGmUrGmCrUA
miR-30c-5p	rU*rG*rUrArArArCrArUrCrCrUrArCrArCrUrCrUrCmAmGrC	mUmGrAmGrAmGrUmGrUmArGmGrAmUrGmUrUmUrAmCA
miR-124-3p	rU*rA*rArGrGrCrArCrGrCrGrGrUrGrArArUmGmCrC	mCmArUmUrCmArCmCrGmCrGmUrGmCrCmUrUA
miR-135a-5p	rU*rA*rUrGrGrCrUrUrUrUrUrArUrUrCrCrUrArUrGmUmGrA	mAmCrAmUrAmGrGmArAmUrAmArAmArAmGrCmCrAmUA
miR-138-5p	rA*rG*rCrUrGrGrUrGrUrUrGrUrGrArArUrCrArGrGmCmCrG	mGmCrCmUrGmArUmUrCmArCmArAmCrAmCrCmArGmCU
miR-206-3p	rU*rG*rGrArArUrGrUrArArGrGrArArGrUrGrUrGmUmGrG	mAmCrAmCrAmCrUmUrCmCrUmUrAmCrAmUrUmCrCA
miR-218-5p	rU*rU*rGrUrGrCrUrUrGrArUrCrUrArArCrCrAmUmGrU	mAmUrGmGrUmUrAmGrAmUrCmArAmGrCmArCmAA
Control	rC*rG*rCrGrArCrUrArUrArCrGrCrGrCrArArUrArUmGmGrU	rAmCrCmArUmArUmUrGmCrGmCrGmUrAmUrAmGrUmCrGmCG
**Quantitative real time PCR DNA primers**		
**Gene**	**Forward**	**Reverse**
Cfl2	TCTCGTCCCAGTGCCACCGA	ACTCCAGATGCCATAGTGCCCGC
Hprt	CTGGTTAAGCAGTACAGCCCCAAA	TGGCCTGTATCCAACACTTCGAGA
Vim	AAATGGCTCGTCACCTTCGT	AGAAATCCTGCTCTCCTCGC
Ptbp1	ATTCCGTGTGGTCACAGACA	GTCACTGGAAGGAGCTCAGG
Mdk	CCAGGAGACCATCCGCG	TCCTTTTCCTTTCTTGGCTTTG
miR-142-3p	TGTAGTGTTTCCTACTTTATGGA	Universal primer (Qiagen)
miR-9-5p	TCTTTGGTTATCTAGCTGTATGA	Universal primer (Qiagen)
miR-10a-5p	TACCCTGTAGATCCGAATTTGTG	Universal primer (Qiagen)
miR-29a-3p	TAGCACCATCTGAAATCGGTTA	Universal primer (Qiagen)
miR-30c-5p	TGTAAACATCCTACACTCTCAGC	Universal primer (Qiagen)
miR-124-3p	TAAGGCACGCGGTGAATGCC	Universal primer (Qiagen)
miR-135a-5p	TATGGCTTTTATTCCTATGTGA	Universal primer (Qiagen)
miR-138-5p	AGCTGGTGTTGTGAATCAGGCCG	Universal primer (Qiagen)
miR-206-3p	TGGAATGTAAGGAAGTGTGTGG	Universal primer (Qiagen)
miR-218-5p	TTGTGCTTGATCTAACCATGT	Universal primer (Qiagen)
RNU6B (U6)	GATGACACGCAAATTCGTGAA	Universal primer (Qiagen)

### Lentiviruses

Vimentin shRNA lentiviruses were described in^53^. Cells were transduced simultaneously with both versions of sh-Vim lentiviruses, 24 hrs post seeding, and downstream analyses performed at 72 hrs. For overexpression, human Vimentin coding region, was subcloned into FuW-TetO lentiviral vector downstream of tetracyclin response element. A mix of two lentiviruses, one for the expression of Vimentin and the other with FUW-m2rtTA (reverse tetracycline-controlled transactivator), were transduced at MOI = 5, one hour after primary motor neuron seeding. Doxycycline (1 µg/ml, Sigma) was added at 24 hrs, 72 hrs post infection, and downstream analyses performed at 96 hrs.

### Mitochnodria assays

The following chemicals were added, when mentioned: MitoTracker® Deep Red 633 (M22426, Molecular Probes, 50 nM) tetramethylrhodamine, ethyl ester (TMRE, 200 nM) Hoechst (Sigma, 1 μg/mL) 30 min 37 °c. Oligomycin A (Sigma, 1 µM) for 15 min, in 37 °C and analyzed 24 hrs. afterwards.

### Immunostaining and Western Blot analysis

Cells were rinsed with phosphate buffered saline (PBS) 3 time manually or using Biotek EL406 washer/dispenser for automated 384-well microplates, fixed with 4% formaldehyde (ChemCruz^TM^) for 15 min, permeabilized and blocked in 0.1% Triton X–100 (Sigma), 2% BSA for 20/60 min for 384/24 well plate setup, respectively. Incubation with anti-neuronal Class III beta Tubulin (Tuj1) (1:1000, MRB-435P, Covance, 1.5 hrs) and/or anti-ATP5A, (1:500,ab14748, Abcam) or anti- Microtubule-associated protein 2 (MAP2) (1:500, sc-20172) was followed by anti Cy2- conjugated donkey anti-rabbit IgG (1:200, Jackson ImmunoResearch, 1 hr), nuclear staining with 4'6-Diamidino-2-phenylindole dihydrochloride (DAPI, 1 µg/ml,sigma, 5 min.) and 3 cycles of PBS rinsing. For Western blot studies, 30 μg protein extracts were denatured by boiling in × 5 sample buffer (60 mM Tris-HCl pH 6.8, 25% glycerol, 2% SDS, 14.4 mM β-mercaptoethanol, 0.1% bromophenol blue) for 5 min., resolved by 8% SDS-PAGE, 100–120 V, 70 min. and transferred to nitrocellulose membrane (Whatmann, 10401383) at 250 mA, 70 min. Membranes were stained with Ponceau (Sigma-Aldrich, P7170), blocked for 60 min. at Room Temperature (RT) with 5% milk, 0.05% TWEEN-20 and incubated, rocking, with primary antibodies O.N. at 4 °C, namely, monoclonal mouse anti-Vim supernatant (a gift from Benny Geiger, Weizmann Institute of Science) or monoclonal mouse anti-alpha-Tubulin, (DM1A SigmaT9026 1:2000 in 5% Bovine Serum Albumin, 0.02% sodium azide, 5 drops of phenol red in 0.05% PBST). Membranes were washed 3 × 5 min., RT with 0.05% PBST and incubated 60 min. with horseradish peroxidase (HRP)-conjugated anti-mouse secondary antibodies. Membranes were washed 3 × 5 min., processed with EZ-ECL Chemiluminescence detection kit for HRP (Biological Industries, 20-500-120) and visualized by ImageQuant™ LAS 4000 (GE Healthcare Life Sciences). Vim densitometry values normalized to Tubulin in the same lane. Representative data in main Figs 4e and 5b are depicted on blots, shown in full in Sup. Figure 6.

### Imaging

For high content image analysis, eight/two micrographs taken in 24 /384 well plate setups, respectively, using automated fluorescence microscope (ImageXpress Micro and MetaXpress software, Molecular Devices). Motor neuron were defined by positive to Tuj1 staining (Alexa488, FITC channel) and nuclear stain (DAPI). Phenotypic parameters were quantified with relevant MetaXpress High-Content Image Acquisition modules (Neurite Outgrowth, MWCS). Confocal microscopy performed on Carl-Zeiss 710. Mitochondria Live imaging was performed on Nikon Eclipse Ti Spinning disc confocal with Yokogawa CSU X-1, 60X oil-immersion lens, Andor iXON3 EMCCD camera under controlled environment (37 °C, 5% CO2). Axonal transport analysis was carried by analysis of time-lapse images using imageJ or MATLAB, as in^67,68^. For transmission electron microscopy, motor neuron were prepared, following^69^ and electron micrographs were captured with a FEI Tecnai SPIRIT transmission electron microscope (FEI, Eidhoven, Netherlands), operated at 120 kV and equipped with an EAGLE CCD Camera.

### RNA analysis and Next Generation Sequencing

Total RNA was isolated with miRNeasy micro kit (Qiagen), assessed with Nano Drop ND-1000 Spectrophotometer (Peqlab) and reverse transcribed to cDNA. qPCR, performed with SYBR Green (Thermo Fisher Scientific or Qiagen). miRNA/mRNA levels were normalized to U6/hypoxanthine phosphoribosyltransferase 1 (Hprt), respectively. Primer sequences are described in Table 2. Library prep for NGS, following [46] and sequencing performed on Illumina 2500 at 50 bp single read. Fasta files created (Illumina CASAVA-1.8.2 software) and mapped (TopHat2 version v2.0.10)^70^ against the mouse genome, build mm9. Approximately, 85–90% mapping rate was observed. Only uniquely mapped reads determined the number of reads per gene (HTSeq-count script 0.6.1p1)^71^. Differentially expressed genes, were determined by padj < 0.05 and an absolute fold change >2 (DESeq. 2 package v1.4.5)^72^ and hierarchical clustering using Pearson dissimilarity and complete linkage was performed to explore gene expression patterns (Matlab 8.0.0.783).

### Statistical analysis

Analysis was performed manually or with GraphPad Prism 6 for Student’s *t*-test, test of proportion or one way ANOVA with post-hoc Newman-Keuls or one way ANOVA with Duncan’s new multiple range test (MRT), as indicated. Results are given as mean ± standard error of the mean (s.e.m). The null hypothesis was rejected at the 0.05 level (^*^), 0.01 (^**^) or 0.001 (^***^). Non-significant values on statistical test are not mentioned in the figures. Gene Ontology analysis was performed using DAVID^46^.

## Electronic supplementary material

Supplementary Information
