# Supplementary material for: High content image analysis reveals function of miR-124 upstream of Vimentin in regulating motor neuron mitochondria
Source: Sci Rep. 2018 Jan 8;8:59. doi: 10.1038/s41598-017-17878-x (PMC5758812; doi:10.1038/s41598-017-17878-x)
Supplement: Supplementary file 1 — Supplementary Information [file 41598_2017_17878_MOESM1_ESM.pdf]

Seven supplementary Figures for:

Title:

High content image analysis reveals function of miR-124 upstream of Vimentin in regulating motor neuron mitochondria

Author list:

Tal Yardeni [1,5,6], Raquel Fine [1,6], Yuvraj Joshi [2], Tal Gradus-Pery [2], Noga Kozer [3], Irit Reichenstein [1], Eran Yanowski [1], Shir Nevo [1], Hila Weiss-Tishler [1], Michal Eisenberg-Bord [1], Tal Shalit [4], Alexander Plotnikov [3], Haim M. Barr [3], Eran Perlson [2], and Eran Hornstein [1,7].

Affiliation:

1 Department of Molecular Genetics, Weizmann Institute of Science, Rehovot 76100, Israel
2 Department of Physiology and Pharmacology, Sackler Faculty of Medicine, Tel Aviv University, Tel Aviv, Israel
3 HTS unit, G-INCPM, Weizmann Institute of Science, Rehovot 76100, Israel
4 Bioinformatics unit, G-INCPM, Weizmann Institute of Science, Rehovot 76100, Israel
5 Current address: Center for Mitochondrial and Epigenomic Medicine, Children's Hospital of Philadelphia, Philadelphia, Pennsylvania, USA.
6 These authors contributed equally to the study
7 Corresponding Author: Eran Hornstein. <eran.hornstein@weizmann.ac.il>
1314 Meyer Building Department of Molecular Genetics, Weizmann Institute of Science, Rehovot 76100, Israel

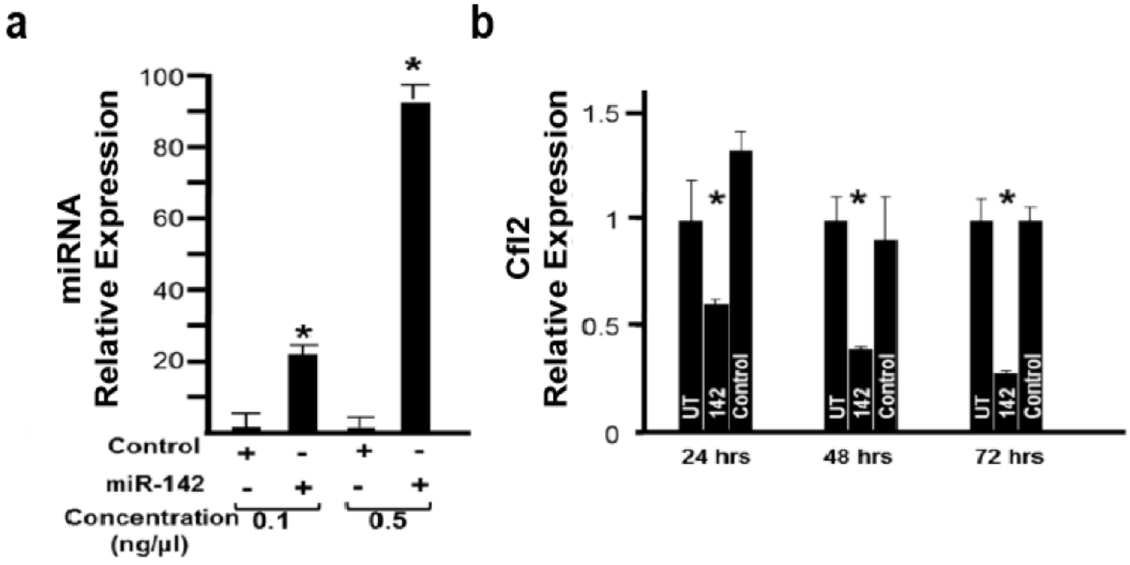

Supplementary Figure 1. The efficacy of miRNA mimics transfection to motor neurons with lipid nanoparticles. (a) miR-142 mimics, encapsulated into lipid nanoparticles from Precision Nanosystems [33], were transfected at (0.1 or 0.5 ng/ µl) to primary motor neurons. miRNA levels were tested 72 hrs after transfection by qPCR, relative to transfection of negative control duplex sequence (IDT NC5) and normalized to U6 levels in the same samples. (b) Downregulation of cofilin-2 (Cfl2), a known target of miR-142 [35], was quantified at 24, 48 and 72 hrs after transfection with miR-142 mimics (0.5 ng/ µl). Cfl2 levels normalized to Hprt in the same samples. Averages ± Standard error of the mean (SEM), Student’s t-test. * P-value < 0.05. UT, un-transfected.

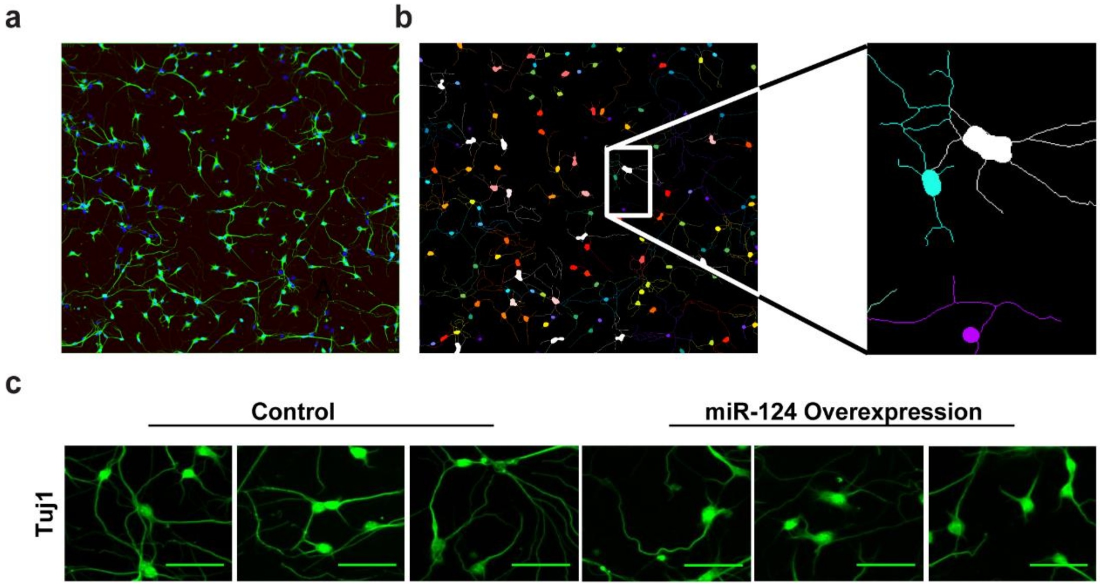

Supplementary Figure 2. High content image analysis. (a) A representative micrograph of a field, showing primary mouse motor neurons, typical of our experimental setup in 384 multi-well plate. Neurons stained with anti Tuj1 antibody and DAPI counterstains nuclei, captured using ImageXpress Micro XLS Wide-field High-Content Analysis System (Molecular Devices). (b) Demonstration of automated feature analysis with MetaXpress2 software (Molecular Devices), depicting individual neurons by different virtual colors and larger power inset on far right. (c) Representative micrographs from three different fields, depicting primary motor neurons transfected with negative control duplex sequence (IDT NC5), or with miR-124 overexpression mimics. Scale Bar 50µm. Routine quantification in 384 multi-well plates considered 500 Tuj1+ neurons quantified per field, 2 fields/well and 6 wells per treatment in five independent experimental repeats. Data collected from >30,000 Tuj1+ neurons per treatment.

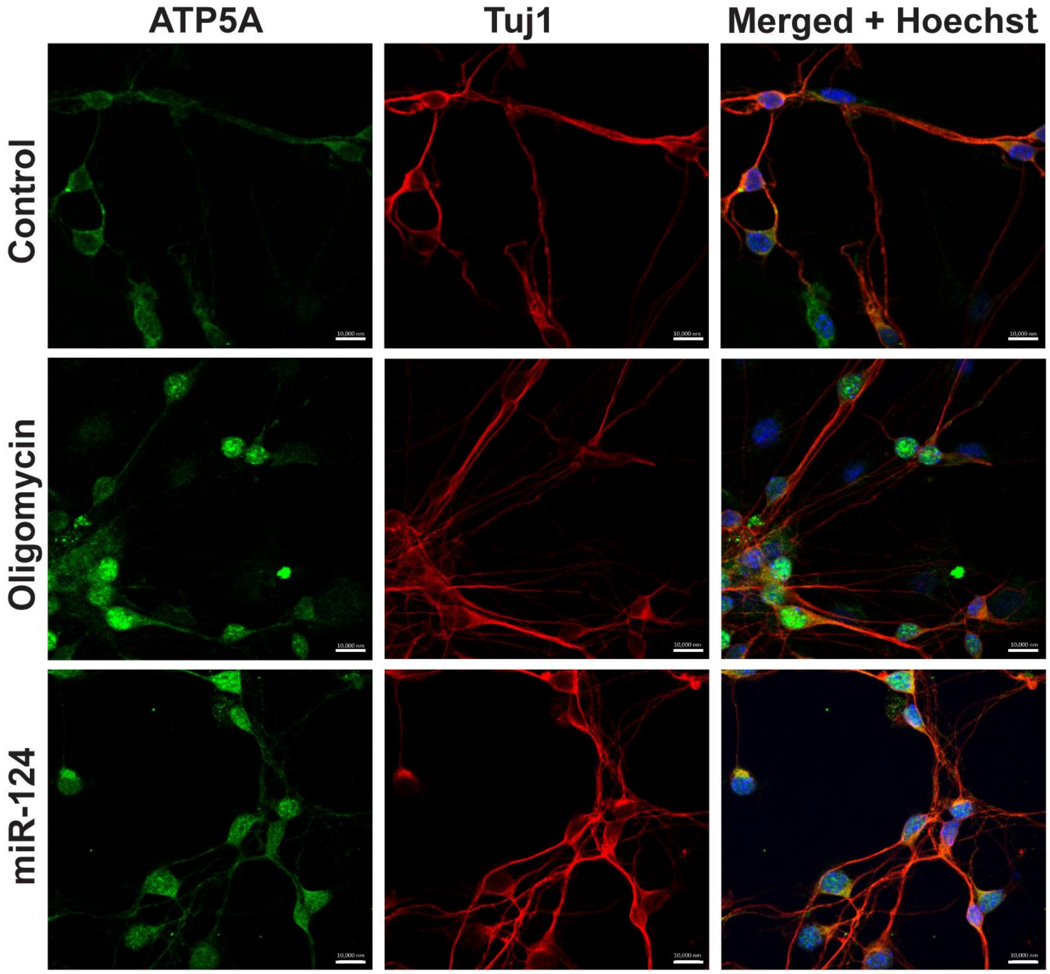

Supplementary Figure 3. miR-124 overexpression impacts mitochondrial localization. Immunofluorescent detection of a mitochondrial marker (anti-ATP5A, green) and neuronal marker (anti-Tuj1, red) in soma and axons. Merged channel includes nuclear staining (DAPI, blue). Primary motor neurons, 72 hrs. post transfection with control oligos or with miR-124 mimics, or treated with Oligomycin A (1µM), an inhibitor of mitochondrial ATP-synthase. miR-124 overexpression or treatment with Oligomycin A, resulted in perinuclear distribution of mitochondria. Scale bars, 10 µm.

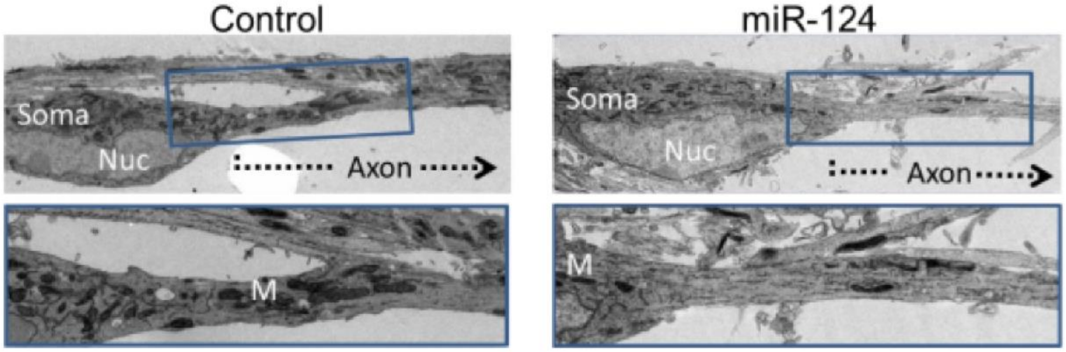

Supplementary Figure 4. Ultrastructural study of motor neurons reveals that miR-124 impact mitochondria localization. Ultrastructural analysis reveals reduction of mitochondria in primary motor neuron axon after miR-124 overexpression, relative to control. Soma, nucleus (Nuc) and mitochondria (M) are depicted. Axon growth direction mentioned by a dashed arrow. Blue rectangles in upper micrographs are enlarged in lower panels.

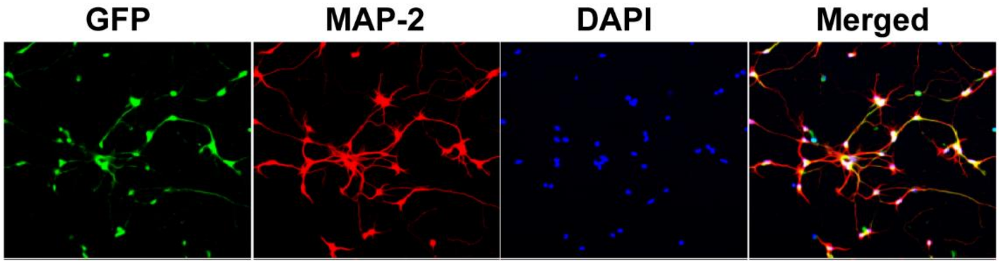

Supplementary Figure 5. Calibration of lentivirus efficacy on mouse primary motor neuron. Mouse primary motor neuron transduced with lentivirus that drives the expression of GFP (transduced at multiplicity of infection (MOI) =1). GFP auto-fluorescence (green), anti-Map2 (Red), DAPI (Blue). GFP - Map2 co-localization demonstrates neuron transduction efficiency of >80%.

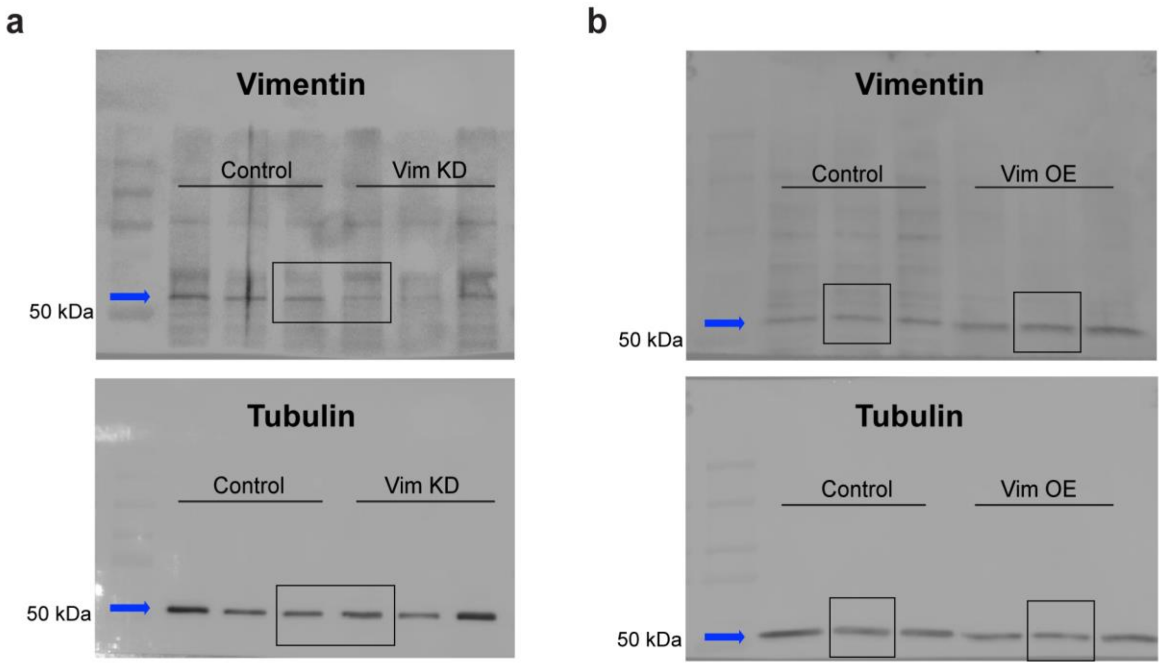

Supplementary Figure 6. Western blot analysis of Vim KD and Vim overexpression lentiviruses. Western blot analysis of Vim and Tubulin in cell extracts from primary motor neurons. (a) Vim was knocked down by shRNA lentiviruses [51] or non-targeting shRNA control lentiviruses. Representative data, depicted for main Figure 4e, are delineated by rectangles. (b) Vim was overexpressed by lentiviruses that enables Doxycycline-dependent expression of Vim. Control conditions - without the chemical inducer (Dox). Representative data, depicted for main Figure 5b, are delineated by rectangles. Estimated lentivirus transduction efficacy >80% of motor neurons.

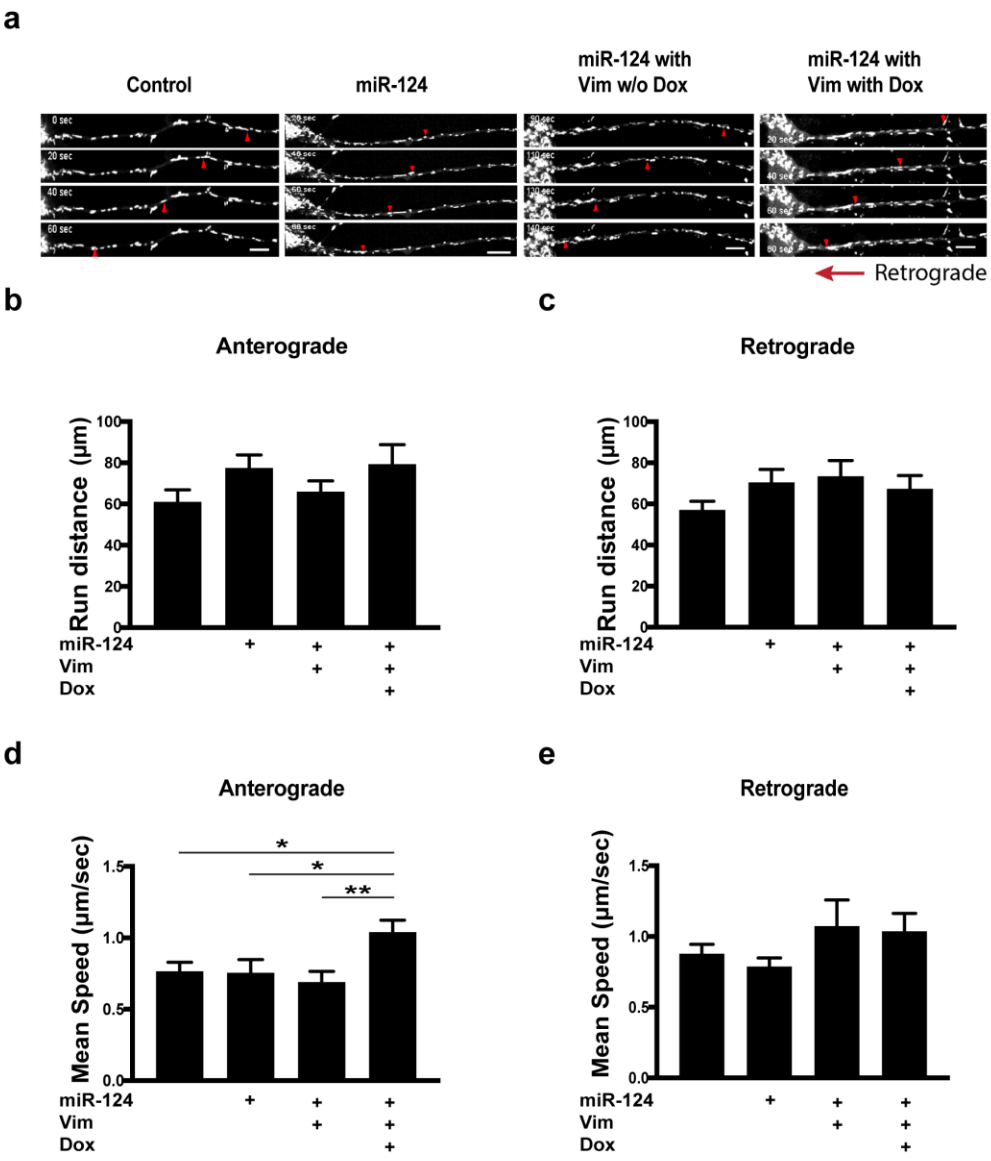

Supplementary Figure 7. Live imaging of mitochondria motility dynamics. (a) Representative captures from mitochondria live imaging from motor axons, complementary to micrographs in Fig 6 A. Red arrows depict retrograde movement of a single, representative, mitochondrion. Quantification of mitochondria live imaging parameters, including overall (b) anterograde or (c) retrograde run distance and (d) anterograde or (e) retrograde mean speed. Running mitochondria were defined by moving a distance of >10 µm at average speed > 0.2 µm/sec. Paused mitochondria are a subpopulation of the running mitochondria, arrested on the same location for ≥3 frames in succession. Horizontal scale bar - 10 µm. Vertical scale bar - 60 seconds. Student's t-test. P-value * < 0.05; ** < 0.01.
